# Affinity Sensors for the Diagnosis of COVID-19

**DOI:** 10.3390/mi12040390

**Published:** 2021-04-02

**Authors:** Maryia Drobysh, Almira Ramanaviciene, Roman Viter, Arunas Ramanavicius

**Affiliations:** 1 State Research Institute Center for Physical and Technological Sciences, Sauletekio ave. 3, LT-10257 Vilnius, Lithuania; mariadrobysh@gmail.com; 2 NanoTechnas–Center of Nanotechnology and Materials Science, Faculty of Chemistry and Geosciences, Vilnius University, Naugarduko str. 24, 03225 Vilnius, Lithuania; almira.ramanaviciene@chf.vu.lt; 3 Center for Collective Use of Scientific Equipment, Sumy State University, 31, Sanatornaya st., 40018 Sumy, Ukraine; 4 Institute of Atomic Physics and Spectroscopy, University of Latvia, Jelgavas Street 3, LV-1004 Riga, Latvia

**Keywords:** COVID-19, SARS-CoV-2 virus, RNA analysis, bioelectrochemistry, biosensors, electrochemical immunosensors, antigen-antibody interaction, immune complex, molecularly imprinted polymers (MIPs), surface modification by immobilization of biomolecules

## Abstract

The coronavirus disease 2019 (COVID-19) outbreak caused by severe acute respiratory syndrome coronavirus 2 (SARS-CoV-2) was proclaimed a global pandemic in March 2020. Reducing the dissemination rate, in particular by tracking the infected people and their contacts, is the main instrument against infection spreading. Therefore, the creation and implementation of fast, reliable and responsive methods suitable for the diagnosis of COVID-19 are required. These needs can be fulfilled using affinity sensors, which differ in applied detection methods and markers that are generating analytical signals. Recently, nucleic acid hybridization, antigen-antibody interaction, and change of reactive oxygen species (ROS) level are mostly used for the generation of analytical signals, which can be accurately measured by electrochemical, optical, surface plasmon resonance, field-effect transistors, and some other methods and transducers. Electrochemical biosensors are the most consistent with the general trend towards, acceleration, and simplification of the bioanalytical process. These biosensors mostly are based on the determination of antigen-antibody interaction and are robust, sensitive, accurate, and sometimes enable label-free detection of an analyte. Along with the specification of biosensors, we also provide a brief overview of generally used testing techniques, and the description of the structure, life cycle and immune host response to SARS-CoV-2, and some deeper details of analytical signal detection principles.

## 1. Introduction

The spreading of severe acute respiratory syndrome coronavirus 2 (SARS-CoV-2), which is causing coronavirus disease 2019 (COVID-19) was declared as a global pandemic in March 2020. The main threat of the pandemic is the overloading of the health systems. The key tool against infection spreading is decreasing its distribution rate, in particular by monitoring the infected people and their contacts. For the successful control, the primary step is the detection of SARS-CoV-2 in an organism. Hence, the development and introduction of rapid, precise, and sensitive detection methods are required. For a better understanding of the existing detection method principles, it is worth dwelling in more detail on the structure of SARS-CoV-2, its life cycle and the induced host response.

## 2. The Structure of SARS-CoV-2 Virus

The coronavirus SARS-CoV-2 is a spherical structure with a diameter of about 130 nm [1,2,3], its surface is riddled by spikes making the viral particle look like the ‘Sun’s corona’, therefore, similarly looking viruses are named as coronaviruses. Inside the viral structure, a helically symmetrical nucleocapsid containing ssRNA, which is a genetic information carrier of this virus, is located. The SARS-CoV-2 has a typical for the coronaviruses (CoVs) genome, which is by about 80% and 50% similar to that of known SARS-CoV and middle east respiratory syndrome coronavirus (MERS-CoV), respectively [4]. The genome is including no less than ten open reading frames (ORFs). The virus replicase-transcriptase complex, which is formed by two large polyproteins, is encoded on the 5′-terminal two-thirds of the genome ORF1a/b, while the entire part of the genome encodes four key structural proteins, that are, spike (S), envelope (E), nucleocapsid (N) and membrane (M) proteins (Table 1). These proteins play a crucial role, primarily in the formation of viral particles, and are taking part in other stages of the SARS-CoV-2 life cycle [4]. The S-protein, a large transmembrane homo-trimer (∼150 kDa), which consists of two subunits, namely, S1 and S2 [4,5,6] and is responsible for the virus attaching to a host cell, followed by fusion and infection [7,8]. An attachment to a host receptor performs through the receptor-binding domain (RBD) in the S1 subunit and then fusion of the viral and host membranes takes place through the S2 subunit [9,10,11,12]. The E-protein (∼8–12 kDa) is a transmembrane and also the smallest one. The smaller part of the E-protein, which is expressed in the infected host cells, is forming the viral overall envelope, while the larger part of this protein is involved in viral association and maturation [13,14]. The N-protein is bound with viral ssRNA, and it is responsible for virion formation [15]. The N-protein consists of a monomeric N-terminal domain (NTD) with a mass value of about 15.4 kDa, and a dimeric C-terminal domain (CTD) with a mass of ~28.7 kDa, in such a way that both are needed for the ssRNA binding [15,16,17]. The M-protein (∼25–30 kDa) is responsible for the shaping of the viral envelope [18]. The M-protein is characterized by the cooperation with 3 other main proteins of SARS-CoV-2. The interaction between S- and M-proteins helps to hold the S-protein in the endoplasmic reticulum (ER)-Golgi intermediate compartment (ERGIC)/Golgi complex followed by its integration into novel virions [19]. The interplay of M- and N-proteins leads to the stabilization of the N-protein/ssRNA complex (nucleocapsid) and the internal core of virions [20]. The complex of M- and E-proteins is involved in the process of viral envelope formation, and it is responsible for the creation and extrication of virus-like particles (VLPs) [21].

## 3. Life Cycle of SARS-CoV-2 Virus

Primarily SARS-CoV-2 infects the respiratory system with the following replication of the virus in the alveoli, causing rupture of the alveolar vessels with the sub-sequent virus leakage into the bloodstream [22]. Further SARS-CoV-2 might attack the cells of other organs characterized by high expressing angiotensin-converting enzyme 2 (ACE2) receptors, such as absorptive enterocytes from the ileum and colon [23], cholangiocytes [24], myocardial cells, kidney proximal tubule cells, and bladder urothelial cells [25].

SARS-CoV-2 virus attaches to the host cell by the RBD within the S1 subunit of S-protein and a cellular receptor. In the case of SARS-CoV-2, angiotensin-converting enzyme 2 acts as the host cell receptor [4,26]. Furthermore, the virus is entered into the host cell cytosol through the acid-dependent proteolytic splitting of the S-protein, which leads to the merging of the viral and host membranes with subsequent viral genome injection into the cytoplasm [4]. The translation of the replicase gene from the virion genomic RNA is the next stage. Proteases encoded by CoVs cleave the replicases polyproteins, and then nonstructural proteins (nsps) form the replicase-transcriptase complex (RTC) for RNA synthesis. During the viral RNA synthesis, genomic and subgenomic RNAs are produced, the last one acts as mRNAs for the structural and accessory genes. Afterward, the S, E, and M-proteins are translated and placed into ER, then the proteins are transferred through the secretory pathway into the endoplasmic-reticulum–Golgi intermediate compartment [27,28]. There, N-protein forms the nucleocapsid of the viral genome, which further sprouts into the membrane with subsequent formation of the virus [15], whereas the M-protein manages the protein-protein interactions necessary for the formation of the viral particle. After formation, vesicles transfer the virions to the cellular surface, where exocytosis takes place [4].

## 4. Humoral Immune Response towards SARS-CoV-2 Virus Infection

The entry of the SARS-CoV-2 into the host triggers an immune response to eliminate the virus, which is initially represented by innate immune system cells, for example, macrophages [29]. Sequential chain stimulation of the different immune cells leads to the inducing of the humoral immune responses by expressing antigen-specific antibodies. The antibodies expressed are mostly immunoglobulins M (IgM) and G (IgG), which are a unique marker for the presence of coronavirus [30]. IgM peak appears approximately after 2–5 weeks, while IgG peak appears later, approximately after 3–7 weeks from post-symptom onset and remains relatively stable up to 105 days [31,32]. Structural proteins of SARS-CoV-2, namely, the S- and N-proteins, act as antigens for specific binding to antibodies [33].

## 5. Common Diagnostic Types

**Figure 1:**
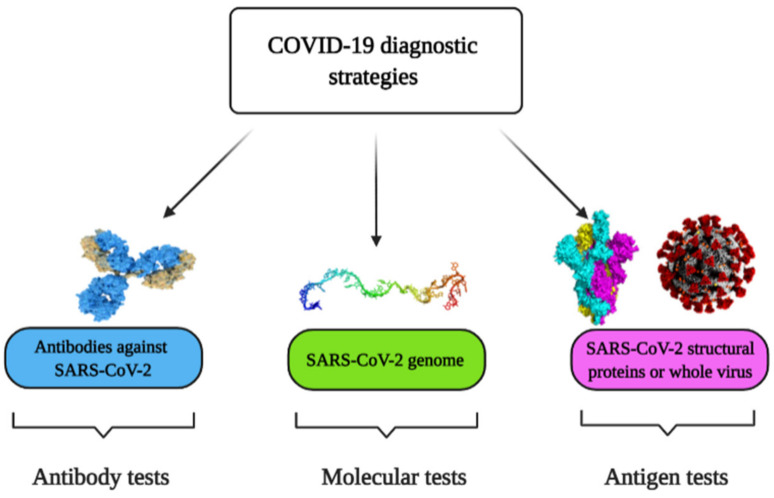
Coronavirus disease 2019 (COVID-19) diagnostic strategies.

There are three general strategies currently used to detect SARS-CoV-2 and to diagnose COVID-19, namely, molecular tests based on the determination of viral RNA, antigen tests based on the determination of viral proteins, and antibody tests based on the determination of specific antibodies against viral proteins (Figure 1).

### 5.1. Molecular Methods for the Detection of SARS-CoV-2 Virus

Molecular-based approaches enable determining whether SARS-CoV-2 infection is currently present and active in the host organism. To date, the ‘gold standard’ is based on the application of Reverse Transcription Polymerase Chain Reaction (RT-PCR) [34], which is aimed at the detection of viral RNA. The method is based on the reverse transcription of the RNA into complementary DNA (cDNA) followed by cDNA amplification and detection by quantitative RT-PCR [35]. Signal registration might be performed by tracking the active and ongoing status of the reaction (real-time) or through post-reaction analysis. Detection time for quantitative RT-PCR is around 1 h with limit of detection (LOD) 0.689 copies/µL [36]. The main limitations of this approach are the requirement for expensive equipment and the need for highly qualified personnel [37]. Another commonly used molecular method is the Reverse Transcription Loop-Mediated Isothermal Amplification (RT-LAMP). LAMP is an improved amplification method by employing DNA polymerase enzyme coupled with several diverse primers, each recognizing dissimilar regions of the target DNA [38]. RT-LAMP assay enables performing transcription and amplification at the same time by blend LAMP-approach based on enzyme–reverse transcriptase [37]. RT-LAMP requires around 30 min per test with LOD 200 copies/reaction [39]. The advantages of RT-LAMP methods are based on the cost reduction of the test by avoiding some steps, which usually are required for the quantitative RT-PCR method and reducing the duration of analysis and the probability of contamination. The disadvantage of this approach is the complicated design of the required primers [37]. One more isothermal amplification method is based on recombinase polymerase amplification (RPA), which involves two primers that bind a double-stranded template using single-stranded binding proteins and recombinase, followed by extension with DNA polymerase [40]. Recently, reverse transcription RPA (RT-RPA) based assay for SARS-CoV-2 detection was reported characterized by test time less than 20 min and LOD 5 copies/μL [41]. Consider also one of the most recent molecular SARS-CoV-2 detection methods, which is based on the Clustered Regularly Interspaced Short Palindromic Repeats (CRISPR)-based approach. The key principle of this approach is the use of Cas12/Cas13 enzymes, which initially were determined as components of the ‘bacterial immune system’ coupled to RNAs with subsequent specific binding to certain regions of target DNA or RNA [42]. There are two commonly used CRISPR-based SARS-CoV-2 detection techniques: DNA Endonuclease-Targeted CRISPR Trans Reporter (DETECTR) and STOPCovid (SHERLOCK Testing in One Pot COVID). The first technique is targeted to the E-gene and N-gene of SARS-CoV-2 and employs the Cas12a enzyme, while the second one is aimed at the N gene and utilizes the Cas12b enzyme. The essence of the approach is common: the first step is based on the employment of RT-LAMP, followed by utilizing Cas12 enzymes for cleavage of target biomolecules and signal detection by the lateral flow or fluorescent assay [42,43]. Detection times/LODs for DETECTR and SHERLOCK for SARS-CoV-2 are ∼30 min/10 copies/µL [44] and ∼40 min/2 copies/µL [45] correspondingly. A more detailed overview of action DNA-enzyme-based assays, including those based on the action of CRISPR-Cas system, is presented in our previous review article [46]. The simultaneous application of primer-specific amplification and guide RNA-directed detection significantly improved the specificity of the method, however, the limitations of amplification techniques and the activity of the employed Cas enzymes might affect the test results [42].

Hence, based on the description of the listed techniques, it can be concluded that the fastest and at the same time the most sensitive method is RT-RPA. Nevertheless, all of the above mentioned methods have similar limitations that are the laboriousness and time-consuming of the process that requires complex equipment and highly qualified personnel.

### 5.2. The Determination of Specific Antibodies against SARS-CoV-2

The determination of antibodies belongs to serological methods and in contrast to molecular-based approaches, antibody tests enable confirming that the patient was infected by SARS-CoV-2 in the past, thus allowing for monitoring the stages of the disease, and identifying people who already have immunity to this virus. These tests are based on the detection of a host response, namely, the production of antibodies against the SARS-CoV-2 proteins. Lateral Flow Immunoassay (LFIA) is one of the conventional methods for coronaviruses-related diseases, which represents a paper-like membrane strip that contains a sample well, a conjugate pad (contains SARS-CoV-2 antigen-gold conjugates and rabbit antibody-gold conjugates), a test lines (coated with anti-human IgG and IgM antibodies correspondingly), a control line (coated with anti-rabbit IgG antibodies). After the sample addition, specific IgG and IgM antibodies flow by capillary action toward the lines going through the conjugate pad where specific immunoglobulins bind with SARS-CoV-2 gold conjugated antigens. The formed immune complexes bind with immobilized anti-human IgG and IgM antibodies at the test lines, whereas rabbit gold conjugated antibodies bind to the control line while interacting with immobilized anti-rabbit IgG antibodies. The presented serological method has the main advantage of being suitable to diagnose COVID-19 at the different infection stages due to the combined determination of IgG and IgM. Moreover, IgG-IgM LFIA was reported as an accurate assay with a sensitivity of 88.66% and specificity of 90.63% with test time less than 15 min [47]. One more conventional antibody-based test is the Enzyme-Linked Immunosorbent Assay (ELISA). For the analysis, the inner surface of multiwell polystyrene plates was coated with SARS-CoV-2 antigen [48], afterward, the patient sample was added and incubated for an hour. Furthermore, secondary antibodies conjugated with a reported enzyme were added. These secondary antibodies are bound to specific antibodies present in the immune complex with SARS-CoV-2 antigen [49]. After the addition of the ready-to-use substrate to the enzyme bound to the secondary antibody (with 3,3′,5,5′-tetramethylbenzidine as chromogen), the specific antibodies present on the surface were detected from the color alterations [50,51]. ELISA showed a sensitivity of 77.3% and specificity of 100% for IgM while those were 83.3% and 95% respectively for IgG on the fourth day after the disease onset [52]. However, this sensible approach requires 2–5 h for the test [53]. Conventional techniques also include chemiluminescence immunoassay (CLIA), which is a label-based method that uses chemiluminescent tags or enzymatic labels with subsequent addition of a luminol-based substrate that induces a chemiluminescence-based signal, the intensity of which can be registered by luminesce detecting system [54]. An example of CLIA assay, which is applied for COVID-19 diagnosis, is rather rapid (takes just 20 min) and in this test magnetic bead-conjugated recombinant N-proteins are used as the capture agents, alkaline phosphatase-labeled anti-immunoglobulin antibodies–as the detection probes and lumigen APS-5–as the chemiluminescent substrate. This method has revealed a sensitivity and specificity 60.76% and 92.25% for IgM and 82.28% and 97.5% for IgG [55]. It should be noted, that magnetic bead- conjugated recombinant S-protein or open reading frame 1a and 1b (ORF1a/b) proteins specific to SARS-CoV-2 might be used as the capture agents [56]. Common advantages of serological assays are: suitability for clinical application because of low costs, short time-to-results, relative simplicity and ability to scale to very large throughput.

However, antibody tests have several drawbacks, including the individuality of immune response for each patient, and seroconversion delay, which prevents immediate testing of probable cases and increases the possibility of false results.

### 5.3. Methods for the Determination of SARS-CoV-2 Antigens

This type of diagnostic includes the properties of the above-mentioned molecular and antibody tests. Like the molecular assay, the antigen detection method allows defining the presence of a current viral infection, whereas the principle of the test procedure is similar to that of the antibody test and is based on the detection of specific antigen-antibody complexes. For the determination of SARS-CoV-2 N-proteins in nasopharyngeal secretions LFIA and fluorescent immunoassay (FIA) are applied. ‘COVID-19 Ag Respi-Strip (CORIS)’ assay refers to LFIA-based type and it is based on a nitrocellulose membrane technology with colloidal gold nanoparticles conjugated with monoclonal antibodies against SARS-CoV-2 N-protein. The method allows determining the antigen in the sample within 15 min with overall sensitivity of 30.2% and specificity of 100% [57]. In FIA, the evaluation of analytical signal performs using fluorescence microscopy. An example of FIA-based approach is ‘standard F COVID-19 Ag FIA’ test dedicated to the determination of SARS-CoV-2 N-proteins. It represents a test cassette on which a pre-extracted sample interacts with a monoclonal anti-SARS-CoV-2 antibody and, after incubation, fluorescence analyzer reads the intensity of fluorescence, which is induced by the antibody-antigen complex formation. Test time is 30 min with sensitivity around 47% [58]. For the determination of N-protein half-strip lateral flow assay (LFA) [59], fluorescence immunochromatography (FIC) [60] and CLIA [61] are also used.

To summarize, conventional molecular and serological methods have some limitations. In the case of molecular approaches, a long sample proceeding time is needed, which also requires sophisticated and expensive facilities. Although serological assays bypass these disadvantages, they are less sensitive and limited for the determination of COVID-19 infection. Biosensors have the potential to become a modern, portable and sensitive alternative to the existing cumbersome and complicated methods. In this review, we consider several types of affinity sensors, which are currently used or might be potentially used for SARS-CoV-2 detection.

## 6. Affinity Biosensors for COVID-19 Diagnosis

SARS-CoV-2 infection can be identified using affinity biosensors [62]. Several different types of signal transduction systems can be applied, which include electrochemical, optical, piezoelectric and some others. Electrochemical affinity biosensors are the most prevalent in biomedical applications due to their cheapness, ease and facility of mass manufacture [62].

### 6.1. Affinity Biosensors for the Determination of SARS-CoV-2 RNA

**Figure 2:**
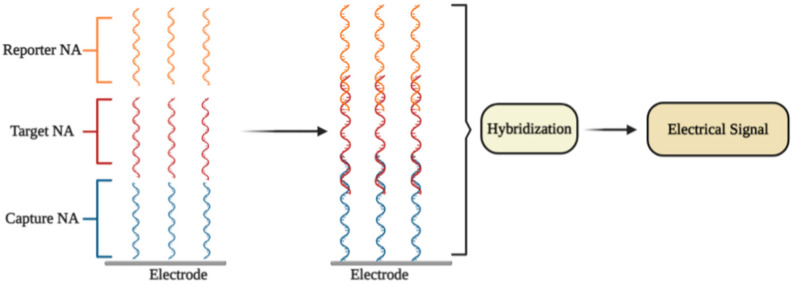
The general principle of electrochemical affinity biosensors for the detection of specific nucleic acid sequences.

An electrochemical DNA/RNA biosensor employs the hybridization of single-stranded nucleic acid (NA) with the complementary strand as a source of the electrochemical signal [63]. The affinity biosensor includes a biorecognition element consisting of the capture NA specifically interacting with the target NA, and the signal transducer where the identification event is transformed into an electrical signal [64] (Figure 2). The detection of specific hybridization of two complementary strands of NA is the key of the affinity biosensor working principle [65,66]. Sometimes additional reporter probes, which are marked with signaling compounds, are used. The reaction of hybridization occurs on an electrode or in a solution [67].

In some electrochemical sensors, NA hybridization [63] includes an electrochemical reaction, which is further used for the quantitation of the detected NA fragment concentration and thus to the concentration of SARS-CoV-2 virus. Electrochemical NA biosensors are classified according to the types of reporter NA (label-free or labeled) and through the signal generation principle (reagent-free or reagent-dependent) [68].

The selective identification of a low amount of DNA and/or RNA copies in specimens is the most important task for electrochemical NA biosensors. The choice of the most efficient signal amplification method is the key aspect that is used to resolve this task. The molecular approaches are classified into (1) NA-based amplification methods (enzyme-mediated isothermal amplification of NA), (2) nanomaterials-based methods (large surface area for the loading of capture NA; nanomaterials as reporter probes), and (3) enzyme-mediated signal amplification (enzymes are connected with NA hybridization system) [68]. Different electrochemical methods are employed for the quantitation of amplified signals, namely, electrochemical impedance spectroscopy (EIS) [69,70], chronoamperometry [71], pulsed amperometric detection [63,72], square wave voltammetry [73], differential pulse voltammetry (DPV) [74], and cyclic voltammetry (CV) [75,76].

#### 6.1.1. DPV-Based Affinity Biosensors

Some researchers presented ultrasensitive DPV-based detection technology using calixarene functionalized graphene oxide for targeting RNA of SARS-CoV-2 [77]. It was affirmed that the technology identifies RNA of SARS-CoV-2 avoiding amplification and reverse transcription stages by employing a portable electrochemical smartphone. The biosensor consists of a capture probe, target sequence, label probe, and an auxiliary probe [78]. The capture probe is complementary to the 5′-terminal of the target sequence, while the label probe is complementary to the 3′-terminal; two different label probe areas have complementary sequences to 5′- and 3′-regions of the auxiliary probe [78,79]. Commonly, each label probe was marked with only one signal compound that led to a low current signal. Hence, it is assumed that transferring the label probe of signaling molecules to other materials or compounds may help to increase the sensitivity [77]. The LOD in the clinical sample is 200 copies/mL, from which it follows that only two copies (10 μL) of all viral RNA copies are needed per analysis. The sensitivity for samples from confirmed COVID-19 patients was 85.5%. [77].

It is worth noting that there are some investigations concerning the potential use of G-quadruplex-based biosensors in COVID-19 diagnosis [80]. G-quadruplex (GQ) is a guanine-rich DNA/RNA sequence, which is folded into four-stranded secondary structures. These structures take part in crucial genome functions such as transcription, replication, and genome stability [81]. Recently, 25 putative G-quadruplex-forming sequences (PQSs) in the genome of SARS-CoV-2 virus were recognized [82]. The PQSs are situated in the ORF1ab, ORF3a, S-, M-, and N-genes of SARS-CoV-2 [80]. Some of the found PQSs are observed in a wide range of coronaviruses, while the main two PQSs, which generate RNA G-quadruplex structures, are strictly observed only in a limited range of viruses. Moreover, a straight interaction between G-quadruplex of coronavirus and viral helicase (nsp13) was obtained by microscale thermophoresis. The results of molecular docking-based modeling suggest that nsp13 alters the G-quadruplex structure. The helicase allows the guanine bases to go out of the guanine quartet planes, therefore, simplifying their unfolding [82]. Thus, RNA G-quadruplex sequences of SARS-CoV-2 could be used for the design of affinity-sensors, which are based on the identification of the viral helicase protein, nsp13 [82].

Fluorescence quenching is a powerful technique for the design of affinity biosensors [83]. One type of biosensor for the determination of enzymes based on fluorescence quenching by G-quadruplex has been reported recently [84]. Guanine at a lower oxidation state can act as the electron donor, while the fluorescence able group acts as an acceptor, which further produces a signal [85].

#### 6.1.2. Plasmonics-Based Affinity Biosensors

The primary concept of plasmonic biosensors is based on the distribution of surface plasmons lengthwise the interface of the thin metallic layer (usually noble metals), and dielectric [86]. This method consists of real-time monitoring changes of the refractive index of the medium surrounding the sensor surface during the interactions between the target biocompound and the immobilized biorecognition element [87,88,89,90]. Most plasmonic biosensors are built on the basics of surface plasmon resonance (SPR) [86,91]. Interactions occur on the surface that is suitable for observation SPR-based signals in two different modes: (1) bulk SPR signal and (2) localized SPR (LSPR) signal. Both effects rely on the refractive index of the ambient media to evoke spectral shifts. Nevertheless, the distinction between SPR and LSPR is defined by the dimensions of applied plasmonic nanomaterials [92].

It was reported that a dual-functional plasmonic biosensor incorporating the plasmonic photothermal (PPT) effect and LSPR sensing transmission enables the development of an alternative approach for SARS-CoV-2 virus detection, where the detection is provided through the hybridization of complementary NA with one NA immobilized on the surface of the gold nanoislands (AuNIs). The LSPR and PPT effects were utilized mutually to increase the signal. The LOD of this assay for the RdRp gene was 0.22 pM. The specificity, the discrimination between the RdRp gene of SARS-CoV and SARS-CoV-2, can be precisely established by onsite PPT improvement on gold AuNIs-based chips [93].

Plasmonic biosensing has technological benefits including the possibility of a combination of SPR with electrochemical, and electroassisted chemiluminescence methods [94]. Moreover, some nanomaterials were applied to establish the optical aperture and reach very sensitive virus identification by SPR method combined with colorimetric and fluorescence determination based approaches [86]. Kinds of plasmonic nanomaterials can alter from metallic nanoparticles and quantum dots to graphene nanostructures [95,96,97,98].

### 6.2. Immunosensors for Determination of SARS-CoV-2 Proteins

#### 6.2.1. Field-Effect Transistor Based Immunosensors

**Figure 3:**
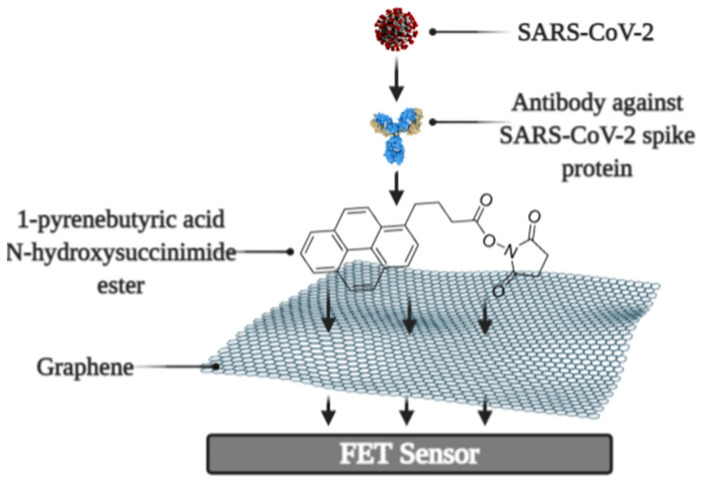
Schematic representation of field-effect transistor-based immunosensor for SARS-CoV-2 detection.

It was reported that a Field-effect transistor (FET)-based biosensor enables the real-time detection of SARS-CoV-2 in clinical specimens. The device was manufactured by covering the graphene plates of the FET with an antibody produced as a response to the SARS-CoV-2 S-protein. The antibody was fixed on the surface of the biosensor by 1-pyrenebutyric acid N-hydroxysuccinimide ester (PBASE) (Figure 3). The cultured virus, antigen protein, and nasopharyngeal swab samples from an infected person have been utilized for the assessment of the efficiency of the immunosensor. It was determined that the FET immunosensor enables the detection of the S-protein at a concentration of 1 fg/mL in PBS and 100 fg/mL in the transport medium, whereas LOD for SARS-CoV-2 was 1.6 × 10^1^ pfu/mL in culture medium and 2.42 × 10^2^ copies/mL in clinical specimens [99].

#### 6.2.2. Quartz Crystal Microbalance Based Biosensors

The quartz crystal microbalance (QCM) can be successfully applied for the development of affinity biosensors [100]. In QCM-based approach, the binding with the viral S-protein occurs on the engineered quartz crystal surface covered by self-assembled monolayer (SAM), and the detection is carried out by QCM. A very simple approach for the determination of proteins is to exploit rather basic surface properties such as hydrophobicity, which is one of the key properties of the working surface of such an analytical system since the increasing the wettability of the surface leads to the increased surface concentration of proteins [101,102]. For this purpose, SAMs with a varied range of hydrophobicity, which is controlled by surface functional groups, were investigated and developed [103]. The SAMs, which have terminal –COOH and –CH_3_ groups, have been shown as the most suitable for the specific and strong binding of SARS-CoV-2 S-protein [104]. The main working principle of the QCM is altering (decreasing) the frequency of the vibrating quartz crystal with the increasing the adsorbed mass [105]. Therefore, QCM-based techniques enable performing sensitive, rapid, and label-free tests [105,106]. 

One more type of affinity biosensor, which is very promising for the determination of virus-induced diseases, is the ultrasound transducer-based immunosensors, e.g.: capacitive micromachined ultrasound transducer (cMUT) was applied in immunosensors for the detection of specific antibodies against some virus proteins [107]. Moreover, the ultrasound-based test allows performing the SARS-CoV-2 virus detection in the gas phase (ultrasonator-produced viral aerosol) [108], while the vast majority of the assays are designed for the solution.

#### 6.2.3. Molecularly Imprinted Polymer Based Electrochemical Affinity Sensors

In affinity sensors, the target protein is detected on the surface of the device, thus the design of the surface with appropriate protein recognition properties is required for the development of such sensors. For this purpose, molecularly imprinted polymers (MIPs) can be very efficiently applied [70,109,110,111,112]. The advantage of molecularly imprinted sensors is that they are cheaper and more stable, and can be based on protein-imprinted polymers such as polypyrrole [113] and some other electrochemically deposited polymers [114,115,116]. Various signal determination methods can be applied in the design of MIP-based sensors, but mostly potentiodynamic electrochemical techniques [113] or QCM-based [100,117] approaches are used for this purpose.

Development and application of MIPs in sensor design is reasonable because MIPs can be developed for small and low molecular weight molecules [75,118]. The efficiency of MIPs for the determination of some virus proteins was also demonstrated [113] and this technology recently was applied for the development of a molecularly imprinted poly-m-phenylenediamine based electrochemical sensors for the detection of SARS-CoV-2 proteins, namely, N-protein [119]. The sensor represented a disposable MIP-modified thin film electrode possessing selectivity to N-protein. Electrochemical signal was observed by DPV and a linear response to N-protein was up to 111 fM with a detection and quantification limit of 15 fM and 50 fM [119].

It should be noted that even some short DNA-based oligomers can be determined by MIP-based sensors [110], which makes MIP-based sensors attractive for DNA and probably for RNA fragment determination. Due to the rather low price of MIPs in comparison to that of antibodies, MIP-related research area is of particular interest and, therefore, MIPs potentially can replace antibodies during the design of various bioanalytical systems and immunosensors.

### 6.3. Ellipsometry and SPR Based Immunosensors

Optical ellipsometry-based techniques have great potential to be applied in the design of various immunosensors [120]. Comparing with other existing methods (ELISA, RT-PCR, indirect fluorescent, western blot) of SARS-CoV detection, the imaging ellipsometry-based approach has established itself as a direct, nondestructive, quick, label-free, simple, and low-cost technique [121].

Recently, spectroscopic ellipsometry (SE) in total internal reflection mode (TIRE) was applied for the monitoring the kinetic of interactions between on SAM-modified gold disk immobilized SARS-CoV-2 N-protein and antibodies against it [122]. TIRE allowed detecting biomolecules mass changes at solid-liquid interface by phase shift measurement. The high sensitivity of SE TIRE was attained with the support of SPR, what enabled the registration of two kinetic curves Ψ(t) and Δ(t) simultaneously [123,124]. It was reported, that antigen-antibody complex is strongly bound and the complex formation has very strict orientation requirements, which was established by meaning of mathematical model building [122]. The main working element of the sensor is the piezoelectric resonator, on which an antigen or antibody is immobilized using SAM-based technology. Incidentally, the application of antibody fragments seems to be a very promising approach for the development of sensors for the determination of virus proteins because it enables increasing the surface concertation of sites that are selective to virus proteins [125,126]

There are some other researches dedicated to the development of biosensors with the potential application for the determination of SARS-CoV-2 infection, based on the antigen-antibody interactions. In order to exploit such interactions, an electrochemical biosensor based on electrode covered with a SAM and specific-antibodies against SARS-CoV-2 proteins, was designed [127]. 

### 6.4. Photoluminescence-Based Immunosensors

**Figure 4:**
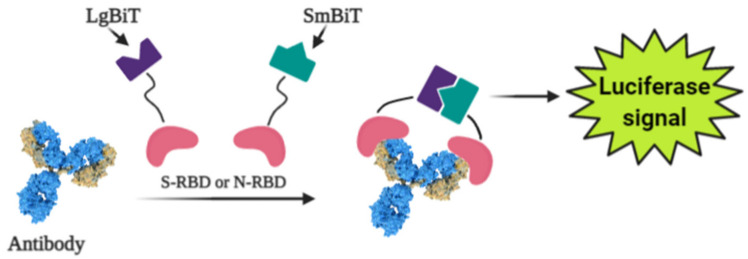
Scheme of the general working principle of split luciferase based immunosensor.

Photoluminescence is a very sensitive technique that can be applied in the design of various affinity biosensors for the determination of pathological cells [128] and virus-induced diseases [129,130,131]. Some researchers designed a split luciferase (spLUC) based antibody test that is showing itself as simple (not need ‘washing’, two-stage of reagent addition, rapid (less than 5 min), reliable (≥98%), low-volume specimen (1 µL for 1 reaction), inexpensive and solution-based quantitative approach to identify antibodies against S- and N- proteins of SARS-CoV-2 [132]. The biosensor was designed by merging small BiT (SmBiT) and large BiT (LgBiT) fragments [133] of Nanoluciferase (NanoLuc) to viral protein antigens. The immunoglobulin has two antigen-binding sites, thus the outcome of incubating 1:1 mixture of SmBiT and LgBiT with serum will be the coupling of one antigen-binding site with LgBiT and another site with SmBiT. The fixing of LgBiT and SmBiT fragments leads to the reduction of NanoLuc enzyme for the following luminescence-based identification (Figure 4).

Sensors based on S- and N-proteins of the SARS-CoV-2 were designed because SARS-CoV-2 infected patients contain antibodies, which are primarily addressed to S- and N-protein epitopes [134,135]. The sensor-based on genetically engineered S-protein containing merged RBD with NanoLuc fragments, whereas for the creation of N-protein-based sensor N-terminal sequence was utilized. The ordinary differential equation modeling was executed to describe the ratio between signal intensity and immunoglobulin concentration and it was shown that there was a linear correlation between the specific antibody concentration and luciferase signal. The sensor showed sensitivity of 89% towards S-protein and 98% towards N-protein [132].

While the existing ELISA-based analysis possesses such disadvantages as laboriousness with numerous washing stages, which complicates point-of-care diagnostics and implementation in regions with limited analytical hardware and reagent sources, the spLUC approach has critical properties that are compliant with all these usages [126]. The reagents used for the spLUC assay were demonstrated to be quite stable to lyophilization for storage and simple transport and rapidly identify immunoglobulins straight from the clinical specimens. The kit containing common pipettes and a portable luminometer is enough for readily setting of the spLUC assay at any care centers despite the infrastructure. The modularity is another benefit of the assay, which can allow accommodating the test to the immune response against almost any infection with known antigens [132].

### 6.5. Determination of Reactive Oxygen Species

It was reported that coronaviruses induce mitochondrial reactive oxygen species (ROS) promotes viral replications in lung host cells [136]. ROS concentrations were significantly enlarged in SARS-CoV^3L^ proexpressing cells [137]. This phenomenon can be explained by the high level of ROS for the activation of SARS-CoV 3a-induced NLRP3 inflammasome [138] because virus infection activates nod-like pyrin domain-containing 3 (NLRP3) family, which is activating the release of ROS from damaged mitochondria [139].

The detector of reactive oxygen species stimulated by COVID-19 is an electrochemical ROS/H_2_O_2_ system [140]. This device includes an integrated portable automatic real-time electrochemical readout board and a sensor, which was made from the multiwall carbon nanotube (MWCNT) on the tip of steel needles. The basic operating principle is the immersion of the electrode into sputum and the latching signals of reactive oxygen species. The intensity of ROS levels, which were released from viral-infected epithelium, were determined by CV.

Unlike other ROS detection approaches, the electrochemical method is rapid (less than 30 s) and can be performed in vivo, without any additional specimen preparation. Electrochemical ROS detection assay was shown as the system operating with lower than 500 μL volumes of aliquots with a rather high accuracy of over 97% [139].

## 7. Conclusions

To date, the scientific community has done a great deal of work studying the properties of the SARS-CoV-2 virus, its spreading, and the effects of the infection on the human body. The most hazardous feature of the virus is the probability of asymptomatic disease, which leads to difficulties in monitoring the spread of the infection as well as an increased probability of fatality due to the late detection of the already developed severe respiratory syndrome. Hence, it is important to have the capacity to diagnose such diseases rapidly to help reduce the distribution of destructive pathogens. Inaccuracies in the time-consuming process of sample preparation and analysis. At the same time, affinity biosensors with similar operating principles can negotiate some of the shortcomings of common approaches.

In this review, we have considered some affinity biosensors used for COVID-19 diagnosis (Table 2). As an analytical signal source, NA hybridization, antigen-antibody interactions, monitoring surface alterations, and changes of ROS levels are employed. The biosensors based on NA hybridization as the signal source were shown as precise, but they still have limitations based on the demand of amplification step and application of specific labels. Among the overviewed sensors, the lowest detection limit is determined for MIP based electrochemical sensor (15 fM). Moreover, MIP-based sensors are more stable in comparison to protein based sensors. ROS detection method is another promising label-free diagnostic method and it is characterized by high sensitivity despite rather low sample volume. Regardless of the unidentified sensitivity, the SE/SPR based technique made it possible to draw important conclusions about the structure of the antigen-antibody complex, as well as to study the kinetics of its formation, which is valuable for the design of new immunosensors. Photoluminescence-based immunosensor is shown as the most sensitive (98% for N-sensor), however it still requires labeling reagents and additional sample preparation steps. The main advantage of QCM and FET based techniques is the ability of real-time tracking of bio-interactions on the working surface.

The commonly used signal registration methods are electrochemical and optical. The examples of affinity sensors reviewed show a clear tendency to design analytical systems that are simple to use due to the elimination of additional steps in probe preparation and the use of auxiliary labeling reagents. These conditions are best met by electrochemical sensors, which detect the interaction of target biomolecules with complementary compounds immobilized on the working surface. Besides, electrochemical biosensors are the most widely used for biomedical purposes due to their cheapness, simplicity, and mass production capability.

It is important to note that, while much work has been done to study the properties of the SARS-CoV-2 and its detection techniques, there is a need to continue to develop and refine diagnostic methods avoiding the shortcomings of these methods, which are already in place, and by exploiting the most significant advantages, which already were achieved by these methods.

## Figures and Tables

**Table 1:**
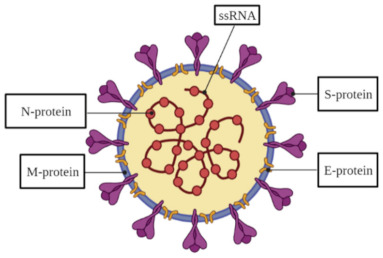
Severe acute respiratory syndrome coronavirus 2 (SARS-CoV-2) structural proteins location, structure, mass and function.

Protein	S-Protein	E-Protein	N-Protein	M-Protein
Subunits	S1 and S2	-	NTD and CTD	-
Mass	∼150 kDa	∼8–12 kDa	∼15.4 kDa (NTD) and ~28.7 kDa (CTD)	∼25–30 kDa
Function	Attachment, fusion and infection of a host cell.	Formation of viral envelope. Association and maturation of the virus.	Virion formation.	Shaping of the viral envelope.

**Table 2:** Summary table of biosensors used for the diagnosis of COVID-19.

#	Biosensor	Biorecognition Element	Signal Source	Registration Methods	Label Need	Immobilization Method	LOD	Sensitivity
**6.1 Affinity biosensors for the determination of SARS-CoV-2 RNA**								
**6.1.1**	Electrochemical	Capture NA	RNA hybrid.	DPV-signal	Label NA	Au/Fe_3_O_4_ NPs	200 copies/mL	85.5%
**6.1.2**	Plasmonics based			PPT+LSPR	Label-free	Au NPs	0.22 pM	-
**6.2 Immunosensors for determination of SARS-CoV-2 proteins**								
**6.2.1**	FET based	Surface properties alterations	Antibody-antigen affinity	FET current response	Label-free	PBASE	242 copies/mL	-
**6.2.2**	QCM based	Surface properties alterations	S-protein binding	Change of QCM resonance frequency	Label-free	SAM	-	-
**6.2.3**	MIP based electrochemical	MIP-modified electrode selective to N-protein	N-protein binding	DPV	Label-free	MIP	15 fM	-
**6.3**	Spectroscopic Ellipsometry	N-protein	Antibody-antigen affinity	TIRE + SPR signals	Label-free	SAM	-	-
**6.4**	Optical	S- or N-protein	Antigen-Antibody affinity	Photoluminescence	SmBiT and LgBiT	-	-	89% (S-sensor) and 98% (N-sensor)
**6.5**	Reactive oxygen species detection	MWCNT electrode	ROS level	CV	Label-free	-	Sputum sample vol. <500 μL	>97%
